# Senolytic agent ABT-263 mitigates low- and high-LET radiation-induced gastrointestinal cancer development in *Apc*^1638N/+^ mice

**DOI:** 10.18632/aging.206183

**Published:** 2025-01-08

**Authors:** Kamendra Kumar, Bo-Hyun Moon, Santosh Kumar, Jerry Angdisen, Bhaskar V.S. Kallakury, Albert J. Fornace, Shubhankar Suman

**Affiliations:** 1 Department of Oncology, Lombardi Comprehensive Cancer Center, Georgetown University Medical Center, Washington, DC 20057, USA; 2 Department of Pathology, Georgetown University Medical Center, Washington, DC 20057, USA; 3 Department of Biochemistry and Molecular & Cellular Biology, Georgetown University Medical Center, Washington, DC 20057, USA

**Keywords:** senescence-associated secretory phenotype, senolytic agent, carcinogenesis, inflammation, β-catenin

## Abstract

Exposure to ionizing radiation (IR), both low-LET (e.g., X-rays, γ rays) and high-LET (e.g., heavy ions), increases the risk of gastrointestinal (GI) cancer. Previous studies have linked IR-induced GI cancer to cellular senescence associated secretory phenotype (SASP) signaling. This study explores the potential of senolytic therapy to mitigate IR-induced GI carcinogenesis. Male *Apc*^1638N/+^ mice were exposed to γ and ^28^Si-ions (69 keV/μm) IR. Two months later, they were treated with the senolytic agent ABT-263 orally for 5 days/week until euthanasia, followed by tumor counting and biospecimen collection at five months post-exposure. Tumors were classified as adenoma or carcinoma by a pathologist. Serum cytokine levels were measured, and the markers of senescence (p16), SASP (IL6), and oncogenic β-catenin signaling were assessed using *in-situ* immunostaining of intestinal tissue. Both low- and high-LET radiation exposure led to an increased frequency of adenoma and carcinoma in *Apc*^1638N/+^ mice, accompanied by increased cellular senescence, acquisition of SASP, and overexpression of BCL-XL protein in a subset of these cells. Furthermore, administration of ABT-263 resulted in the elimination of senescent/SASP cells, a decrease in pro-inflammatory cytokines (TNFRSF1B, CCL20, CXCL4, P-selectin, CCL27, and CXCL16) at the systemic level, and downregulation of β-catenin signaling that coincided with decreased GI cancer development. This study suggests a link between IR-induced senescent/SASP cell accumulation and GI cancer development. It also shows that the senolytic agent ABT-263 can regulate IR-induced inflammatory cytokines and carcinogenic mediators both systemically and in intestinal tissue. These findings support the potential of senolytic intervention to reduce IR-induced GI cancer risk.

## INTRODUCTION

The adverse effects of ionizing radiation (IR) exposure primarily depend on the total absorbed dose, while consideration of linear energy transfer (LET) is important to compare relative differences in the biological effects observed after different IR types [1, 2]. LET specifically refers to the localized energy absorption within the tissue that can distinguish sparsely ionizing (low-LET, such as γ- or X-rays) and densely ionizing (high-LET heavy ions, such as ^28^Si-ions) radiation types [3]. On Earth, human exposure to low-LET IR is common during radiological diagnostic and therapeutic procedures. In contrast, encountering high-LET IR on Earth is relatively less likely but can occur during high-LET cancer radiotherapy with proton or carbon beams, neutron radiation from a radiological dispersal device, or a nuclear disaster involving criticality excursion. It also occurs through exposure to radon gas among miners [4–6]. Notably, high LET radiation exposure to general population can also occur via exposure to radon gas [7], which is produced naturally from the breakdown of uranium in soil, rock, and water that can enter homes through cracks in floors or foundations and can accumulate to high levels, particularly in basements and ground floors where ventilation is limited [8]. Moreover, in deep space (beyond Earth’s magnetosphere), high-LET IR exposure from galactic cosmic radiation (GCR) is an important health risk to astronauts preparing for future interplanetary missions, as spacecraft shielding only provides a modest dose attenuation from high-LET heavy-ion IR and can also emit secondary radiation of varying LET [9]. Nevertheless, exposure to both low- and high-LET IR is known to increase the risk of gastrointestinal (GI) cancer development [10–12].

Among *in vivo* models of radiation-induced GI cancers, *Apc* (adenomatous polyposis coli) gene mutant mouse models have been extensively used due to their remarkable similarity with human colorectal cancers (CRC). Notably, *Apc*^1638N/+^ mouse is a genetic model of spontaneous GI tumorigenesis bearing a point mutation at codon 1638 in one allele of the in *Apc* gene and in C57BL6 background they develop 3-5 tumors throughout the GI tract and mimic sporadic human CRC [10, 11, 13]. Using *Apc*^1638N/+^ mice, it has been demonstrated that exposure to high-LET IR results in a much higher frequency of GI tumors and cancer incidence compared to an equivalent dose of γ-rays [14–16]. Further studies using mathematical and biophysical modeling also suggest a greater risk of high-LET IR-induced GI-cancer and associated mortality risk for astronauts [10]. Therefore, developing a pharmacological mitigator is essential for the prevention of GI cancer incidence among individuals exposed to terrestrial IR as well as astronauts planning to undertake deep space missions.

Both low- and high-LET radiation have been implicated in promoting carcinogenesis through multiple pathways, including DNA damage, chronic cellular stress, and inflammation [12, 14, 16]. Recent studies suggest that one of the critical mechanisms linking IR exposure to cancer development is cellular senescence, a state of irreversible cell cycle arrest accompanied by a distinct secretory phenotype known as the senescence-associated secretory phenotype (SASP) [17–19]. While cellular senescence functions as a tumor-suppressive mechanism by preventing the proliferation of damaged cells [18, 20], accumulating evidence indicates that IR-induced senescence, facilitated by high expression of p16, not only halts cell proliferation but also drives the SASP, thereby influencing tissue homeostasis, promoting inflammation, and tumorigenesis via releasing SASP factors consisting of various pro-inflammatory cytokines, growth factors, and proteases [21–24]. Mechanistically, differential induction of cellular senescence, acquisition of SASP, and subsequent activation of pro-inflammatory and oncogenic β-catenin signaling in mouse GI epithelial cells after low- and high-LET IR-exposure have been reported earlier [16, 17, 25, 26]. Notably, mice exposed to high-LET IR displayed approximately 8 to 15 times greater accumulations of senescent cells and approximately 20 times greater acquisition of SASP, relative to the same dose of γ-rays [14, 16, 17]. In addition to localized GI-tissue specific effects, total body IR exposure induces an accelerated aging phenotype involving multiple tissues marked by a remarkable increase in inflammatory SASP factors at the systemic level [27–29]. SASP factors, including inflammatory cytokines such as interleukin-6 (IL6), tumor necrosis factor receptor superfamily member 1B (TNFRSF1B), Chemokine (C-C Motif) Ligand 20 (CCL20), Chemokine (C-X-C Motif) ligand 4 (CXCL4), P-Selectin (SELP), Chemokine (C-C Motif) Ligand 27 (CCL27), Chemokine (C-X-C Motif) Ligand 16 (CXCL16), are known to exert pro-inflammatory and tumor-promoting effects [30–41]. Therefore, accumulation of both local and systemic SASP above a threshold is attributed to the overall cancer risk [42, 43]. In this context, the identification of strategies to eliminate senescent cells, a process known as senolysis, is hypothesized to serve as a potential means to mitigate IR-induced GI cancer risk [14, 16], but has not been tested before.

In this study, we present compelling evidence demonstrating the efficacy of the pharmacological senolysis approach using ABT-263 (navitoclax), a well-known senolytic agent that exerts its effects through inhibition of anti-apoptotic BCL (B-cell lymphoma) family proteins (BCL-XL, BCL-2, and BCL-W) [44, 45]. Here we demonstrate that post-IR exposure administration of ABT-263 in a mouse model of human GI cancer, i.e., *Apc*^1638N/+^ mice, not only eliminated IR-induced senescent/SASP cells but also attenuated the pro-inflammatory and oncogenic markers induced by IR, both at tissue and systemic levels. Further, ABT-263 administration resulted in a reduced incidence of GI cancer after exposure to both low- and high-LET IR. Our results offer “proof of concept” for the future use of a pharmaceutical senolytic strategy to reduce the risk of IR-induced GI cancer.

## RESULTS

### Low-LET radiation exposure results in increased intestinal tumorigenesis, coinciding with the increased accumulation of senescent and SASP cells in the mouse intestine

**Figure 1:**
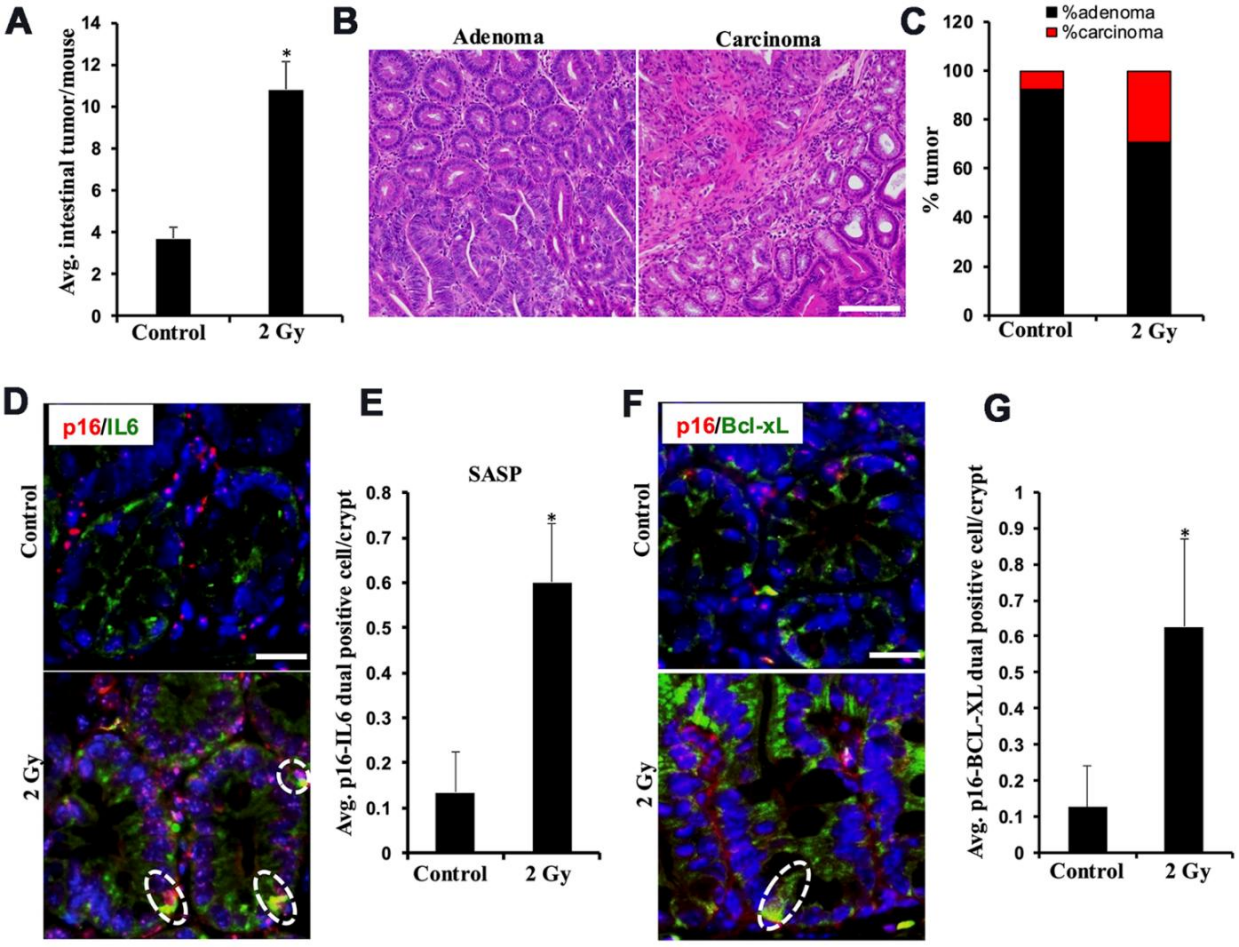
**Low-LET radiation-induced tumor development in *Apc*^1638N/+^ mice is accompanied by an increased number of intestinal cells displaying senescence and SASP.** (**A**) Intestinal-tumorigenesis at 150 days post-exposure (n=10 per group). Data presented as mean ± SEM, and * p<0.05, relative to control animals. (**B**) Representative H&E-stained micrographs of intestinal adenoma and carcinoma. (**C**) Quantification of adenoma and carcinoma as a percentage of total tumors. (**D**) Representative immunofluorescence micrographs of intestinal epithelium showing p16 (red) and IL6 (green) dual positive SASP cells. (**E**) Quantification of p16 and IL6 dual positive cells. Data presented as mean ± SEM, and * p<0.05, relative to control animals. (**F**). Representative immunofluorescence micrographs of intestinal epithelium showing p16 (red) and BCL-XL (green) dual positive senescent cells. (**G**). Quantification of p16 and BCL-XL dual positive cells. Data presented as mean ± SEM, and * p<0.05, relative to control animals.

Low-LET IR exposure to *Apc*^1638N/+^ mice (n=6/group) resulted in a significant increase in overall tumor burden and carcinoma frequency at 5 months post-exposure. Intestinal tumor burden in 2 Gy γ-irradiated *Apc*^1638N/+^ mice was approximately three-fold higher than in the unirradiated control mice (Figure 1A). Histopathological assessment of H&E-stained tumor tissue sections revealed significantly higher increase in the percentage of carcinoma in 2 Gy γ-irradiated mice relative to the control group (Figure 1B, 1C). Previously, IR-induced cellular senescence and activation of SASP signaling have been suggested to play important role(s) in GI-cancer development [14, 16, 17]. Therefore, we assessed cellular senescence and acquisition of SASP in the normal-appearing intestinal tissues, and immunofluorescence-based quantification of p16 and IL6 dual-positive cells in the intestinal mucosa indicated a marked increase in cells displaying SASP in 2 Gy γ-irradiated mouse intestine at 5 months post-exposure relative to the control group (Figure 1D, 1E). Since BCL-2 family proteins are key anti-apoptotic proteins expressed in senescent cells that allow them to survive longer [35], we first identified the most abundant BCL-2 family proteins i.e., BCL-XL (BCL2L1), using mouse and human intestine RNA expression databases (Supplementary Table 1). Further, immunofluorescence-based quantification of p16 and BCL-XL dual-positive cells in the intestinal mucosa suggested that a significant number of IR-induced senescent cells expressed BCL-XL (Figure 1F, 1G). These results revealed that a subset of IR-induced intestinal senescent cells also overexpress BCL-XL, which can be targeted using the senolytic agent ABT-263, known for its BCL-XL inhibitory activity.

### ABT-263 mitigates low-LET radiation-induced intestinal tumor development through elimination of IR-induced SASP cells

**Figure 2:**
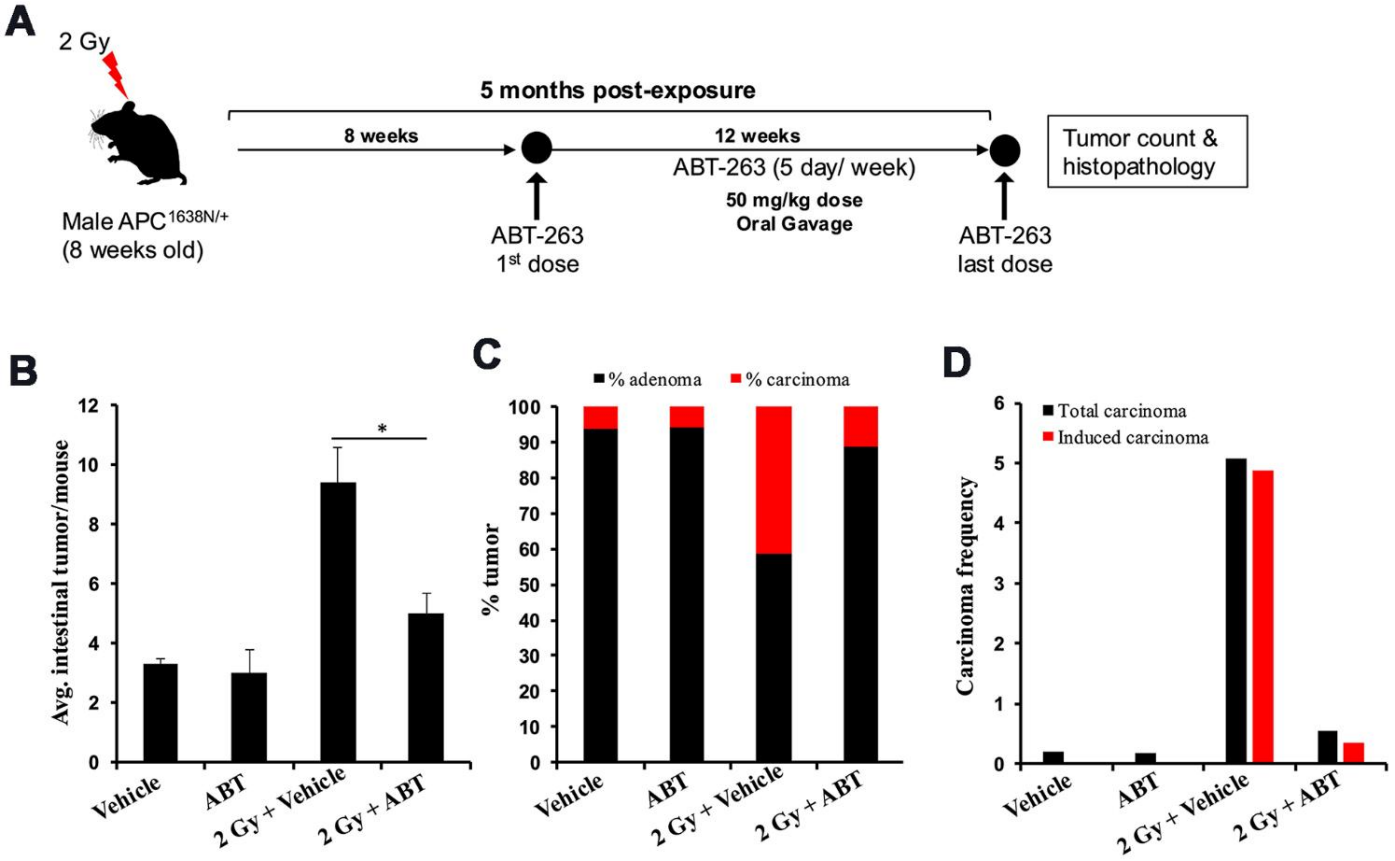
**ABT-263 mitigates γ-induced intestinal tumor development in male *Apc*^1638N/+^ mice.** (**A**) Experimental plan of ABT-263 testing as a mitigator for γ-induced intestinal carcinogenesis. (**B**) Intestinal-tumorigenesis at 150 days post-exposure in vehicle (n=10), ABT-263 only (n=5), 2 Gy + vehicle (n=6), and 2 Gy + ABT-263 (n=6) groups. Data presented as mean ± SEM, and * p<0.05, relative to control animals. (**C**) Quantification of adenoma and carcinoma percentage. For each group, the percentages of adenomas and carcinomas relative to the total number of tumors assessed were calculated. (**D**) Effect of ABT-263 on spontaneous and IR-induced carcinoma frequency. Carcinoma frequency was defined as the number of carcinomas divided by the total tumors assessed. The induced carcinoma frequency was obtained by subtracting the spontaneous carcinoma frequency (from the control group) from that of the irradiated groups.

**Figure 3:**
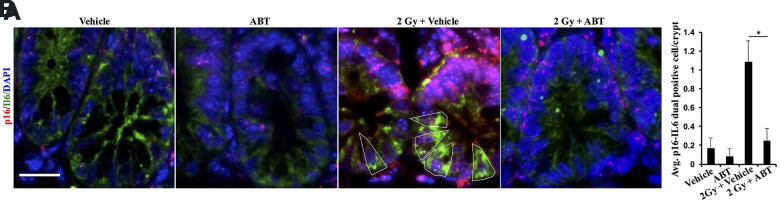
**ABT-263 eliminates SASP cells from the intestinal epithelium of γ-exposed *Apc*^1638N/+^ mice.** (**A**) Representative immunofluorescence micrographs of the intestinal epithelium showing p16 (red) and IL6 (green) dual-positive cells. (**B**) Quantification of p16 and IL6 dual-positive cells. Data presented as mean ± SEM, and * p<0.05, relative to control animals.

We tested ABT-263 (an inhibitor of BCL-XL and a known senolytic agent) for its efficacy to mitigate IR-induced GI-tumorigenesis (Figure 2A). Oral administration of ABT-263 in *Apc*^1638N/+^ mice resulted in a significant reduction in low-LET IR-induced intestinal tumor burden at 5 months post-exposure (Figure 2B). Histopathological assessment of H&E-stained tumor sections also revealed a significant decrease in the percentage of carcinoma in ABT-263 treated group, relative to 2 Gy γ-irradiated mice (Figure 2C). Furthermore, the IR-induced carcinoma frequency in the 2 Gy + Veh group was markedly reduced in the 2 Gy + ABT-263 group (Figure 2D), whereas no significant difference in adenoma and carcinoma frequency was noted between vehicle and ABT-263 treated groups (Figure 2B–2D). These results suggest that ABT-263 can effectively mitigate low-LET IR-induced GI cancer development, while spontaneous GI cancer development was not significantly affected. Immunofluorescence-based co-staining and quantification of p16 (senescence) and IL6 (SASP) dual positive cells in the intestinal mucosa revealed that the reduction in intestinal tumor incidence was accompanied by reduced accumulation of SASP cells in the IR + ABT-263 group, relative to IR + Veh group (Figure 3). These results demonstrate that ABT-263 can effectively remove IR-induced senescent/SASP cells from mouse intestine and can also mitigate IR-induced GI cancer incidence.

### ABT-263 mitigates high-LET ^28^Si-induced tumorigenesis in *Apc*^1638N/+^ mice

**Figure 4:**
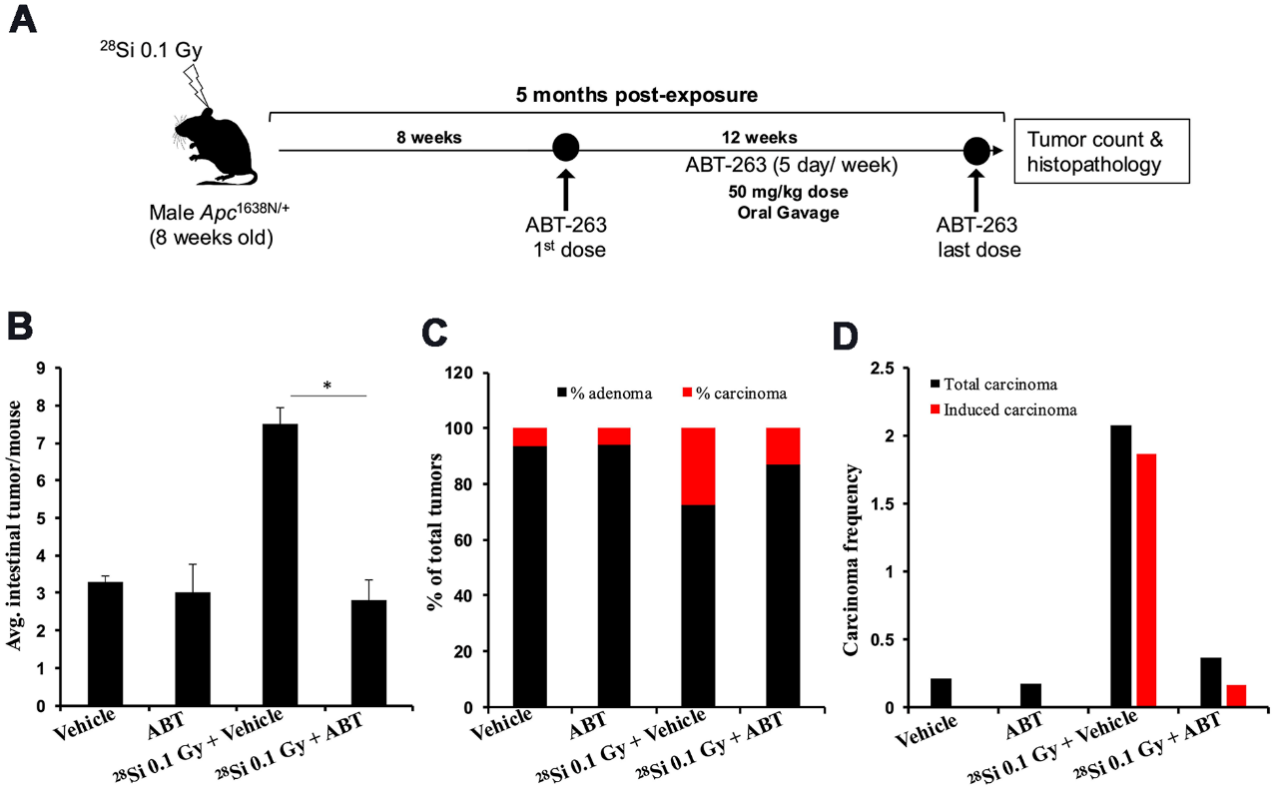
**ABT-263 mitigates high-LET ^28^Si-induced intestinal tumor development in male *Apc*^1638N/+^ mice.** (**A**) Experimental plan of ABT-263 testing as a mitigator for ^28^Si -induced intestinal carcinogenesis. (**B**) Intestinal-tumorigenesis at 150 days post-exposure in vehicle only (n=10), ABT-263 only (n=5), 0.1 Gy ^28^Si + vehicle (n=10), and 0.1 Gy ^28^Si + ABT-263 (n=10) groups. Data presented as mean ± SEM, and * p<0.05, relative to control animals. (**C**) Quantification of adenoma and carcinoma percentage. For each group, the percentages of adenomas and carcinomas relative to the total number of tumors assessed were calculated. (**D**) Effect of ABT-263 on spontaneous and IR-induced carcinoma frequency. Carcinoma frequency was defined as the number of carcinomas divided by the total tumors assessed. The induced carcinoma frequency was obtained by subtracting the spontaneous carcinoma frequency (from the control group) from that of the irradiated groups.

Earlier reports have shown a multifold greater increase in intestinal senescent/SASP cells and intestinal tumor number after high-LET IR relative to low-LET [15, 17, 26]. So, we tested the efficacy of ABT-263 to mitigate high-LET (^28^Si) IR-induced GI-tumorigenesis in *Apc*^1638N/+^ mice (Figure 4A). Oral administration of ABT-263 in *Apc*^1638N/+^ mice resulted in a significant reduction in 0.1 Gy ^28^Si-induced intestinal tumor burden at 5 months post-exposure (Figure 4B). Histopathological assessment of H&E-stained tumor sections also revealed a significant decrease in the percentage of carcinomas in the ABT-263 treated group, relative to 0.1 Gy ^28^Si-irradiated mice (Figure 4C). Furthermore, the IR-induced carcinoma frequency in the 0.1 Gy ^28^Si + Veh group was markedly reduced in the 0.1 Gy ^28^Si + ABT-263 group (Figure 4D), whereas no significant difference in adenoma and carcinoma frequency was noted between vehicle and ABT-263 treated groups (Figure 4B–4D). These results suggest that ABT-263 can effectively mitigate high-LET IR-induced GI cancer.

### ABT-263 dampens high-LET ^28^Si-induced systemic accumulation of pro-inflammatory cytokines in the serum of *Apc*^1638N/+^ mice

**Figure 5:**
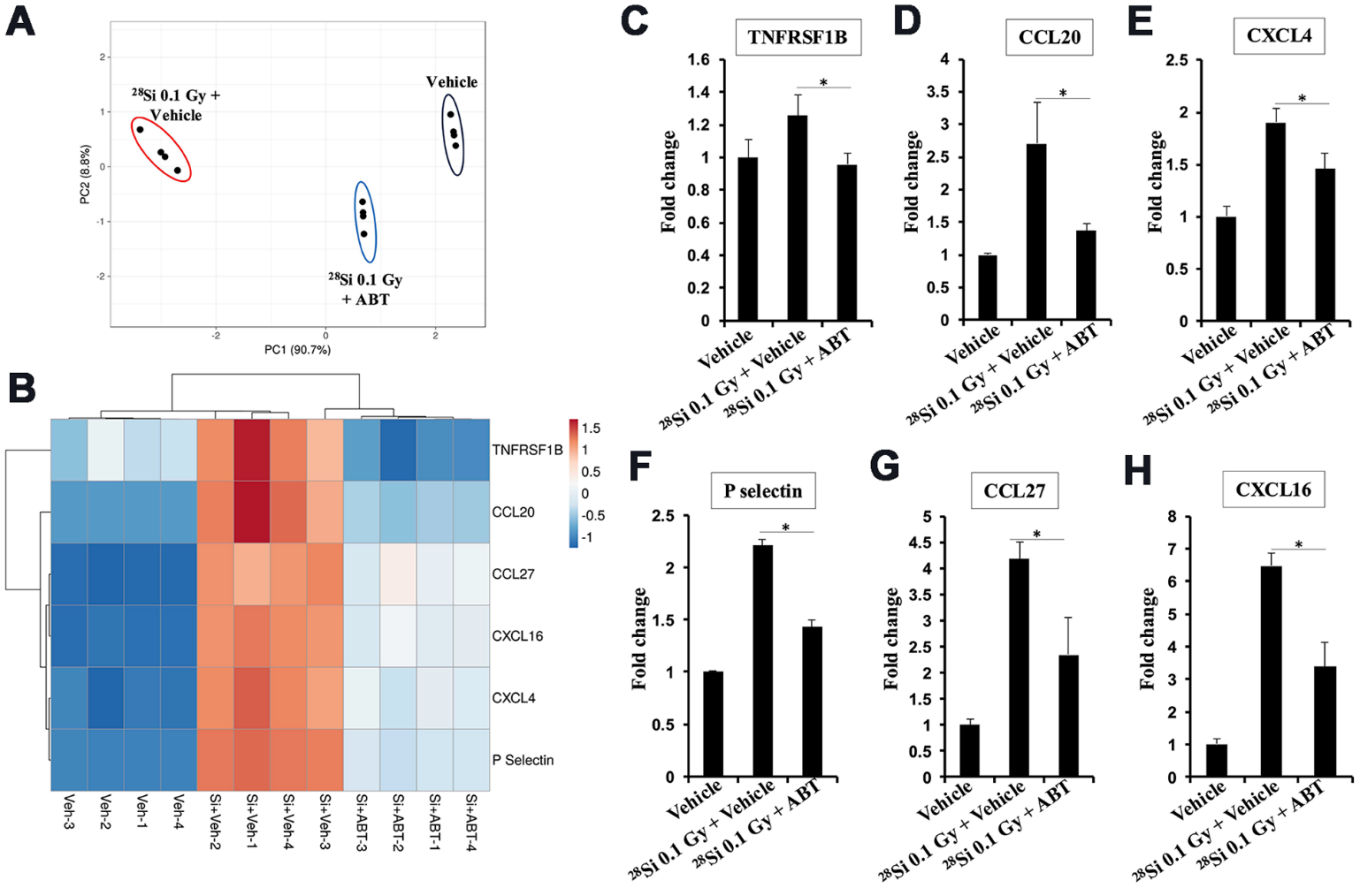
**Effect of ABT-263 on SASP factor expression in the ^28^Si-exposed *Apc*^1638N/+^ mice serum.** (**A**) Principal component analysis (PCA) plot for cytokines showing differential expression. (**B**) Heat map showing differential expression of SASP factor expression in serum obtained from vehicle, ^28^Si + vehicle, and ^28^Si + ABT-263 groups. (**C**) Fold change in serum TNFRSF1B. (**D**) Fold change in serum CCL20. (**E**) Fold change in serum CXCL4. (**F**) Fold change in serum P-selectin. (**G**) Fold change in serum CCL27. (**H**) Fold change in serum CXCL16. Quantitative data presented as mean ± SEM, and * depict a statistically significant difference (p<0.05) between the indicated groups.

We observed a total of six pro-inflammatory and pro-carcinogenic cytokines (TNFRSF1B, CCL20, CXCL4, P-selectin, CCL27, and CXCL16) that increased in *Apc*^1638N/+^ mouse serum after ^28^Si exposure (Figure 5). Principal Component Analysis (PCA) and heatmap analysis (range of measurement -1.25 to 1.7-fold) of differentially expressed cytokines in the control, ^28^Si + Veh, and ^28^Si + ABT-263 groups clearly revealed the efficacy of ABT-263 in reversing the ^28^Si-induced increase in the levels of these cytokines in serum (Figure 5A, 5B). The quantification of individual cytokines from the control, ^28^Si + Veh, and ^28^Si + ABT-263 groups, is presented in Figure 5C–5H. These results suggest that ABT-263 effectively dampened ^28^Si-induced cytokines having a potential role in IR-induced GI cancer development.

### ABT-263 inhibits low- and high-LET radiation-induced oncogenic β-catenin signaling in the intestinal epithelial cells of *Apc*^1638N/+^ mice

**Figure 6:**
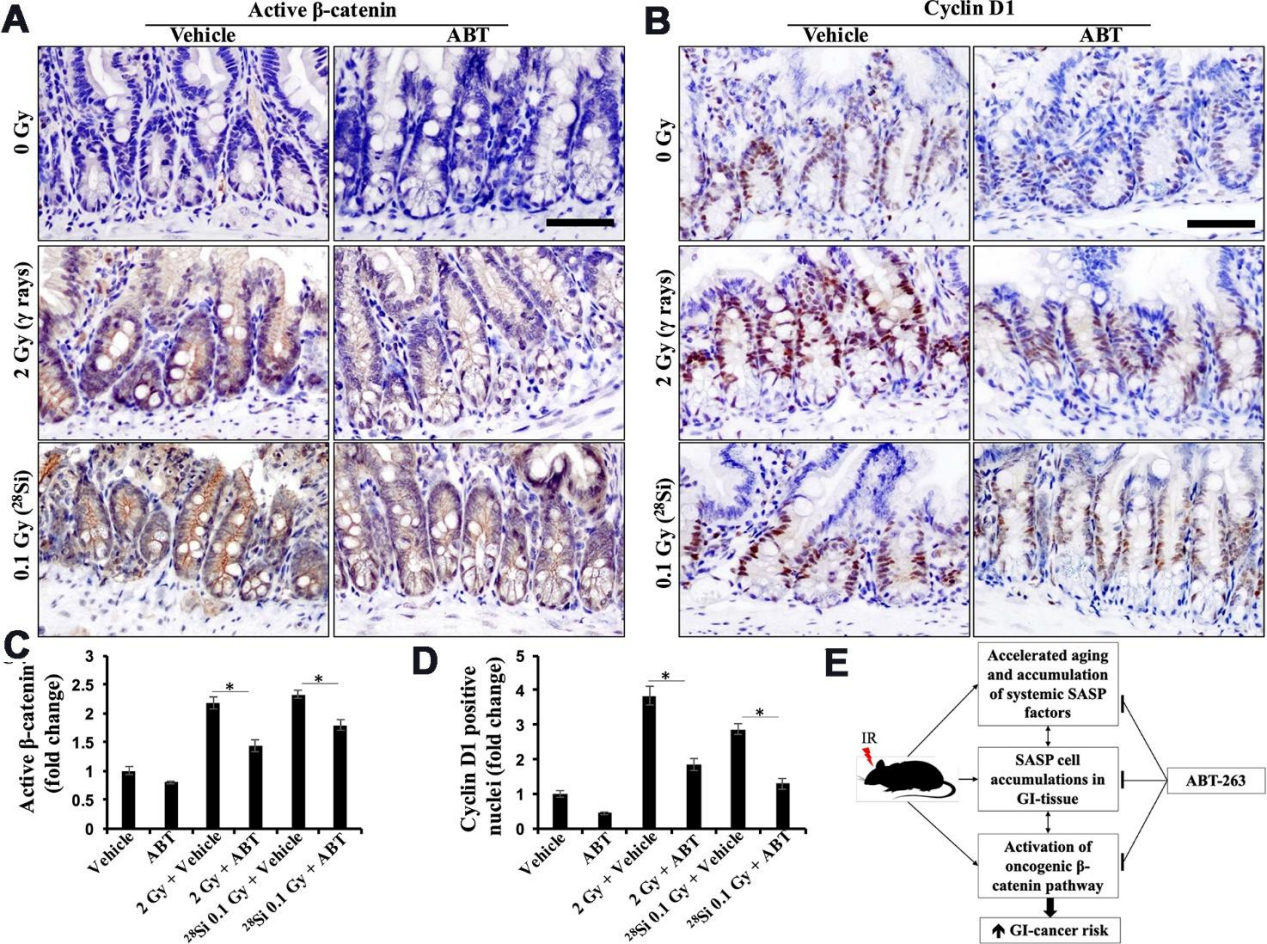
**ABT-263 dampens low- and high-LET radiation-induced oncogenic β-catenin signaling.** (**A**) Representative micrographs of active-β-catenin immunostained intestinal tissue sections. (**B**) Representative micrographs of cyclinD1 immunostained intestinal tissue sections. (**C**) Quantification of active-β-catenin-stained images. Quantitative data presented as mean ± SEM, and * depicts a statistically significant difference (p<0.05) between the indicated groups. (**D**) Quantification of cyclin D1-stained images. Quantitative data presented as mean ± SEM and * depicts a statistically significant difference (p<0.05) between the indicated groups. (**E**) Schematic summary of mechanisms of ABT-263-mediated prevention of IR-induced GI tumorigenesis in *Apc*^1638N/+^ mice.

Given the activation of oncogenic β-catenin signaling in intestinal epithelial cells following exposure to both low and high-LET radiation and its established role in GI cancer development [12, 16, 17, 25, 46], we investigated the impact of ABT-263 on low- and high-LET-induced β-catenin signaling in mouse intestines. Immunohistochemically stained sections of intestinal tissue from both γ and ^28^Si-exposed mice showed significantly higher expression of active β-catenin and its downstream effector cyclin D1. Administration of ABT-263 resulted in a significant decrease in the levels of both active β-catenin and cyclin D1 (Figure 6A, 6B). Quantification of the DAB signal showed significantly higher staining in the intestines of γ and ^28^Si-exposed mice relative to the control and ABT-263-administered groups (Figure 6C, 6D). These results indicate that ABT-263 effectively dampened low- and high-LET IR-induced β-catenin signaling in *Apc*^1638N/+^ mice.

## DISCUSSION

In this study, we tested the plausibility of a pharmacological senolytic approach for mitigation of low- and high-LET IR-induced GI carcinogenesis in *Apc*^1638N/+^ mice. Here, we show that a subset of IR-induced senescent cells acquires the SASP phenotype and also display increased expression of the anti-apoptotic protein BCL-XL. Moreover, oral administration of ABT-263, after IR exposure decreased the incidence of cancer in the mouse intestine, which was accompanied by the elimination of IR-induced senescent/SASP cells and the attenuation of pro-inflammatory and oncogenic signaling at the tissue and systemic levels.

In accordance with earlier reports demonstrating a significantly higher increase in GI tumorigenesis after high-LET IR compared to γ-rays [12, 15], exposure to 0.1 Gy of ^28^Si resulted in a 2.3-fold increase in intestinal tumor frequency. This increase was comparable to the 2.8-fold higher tumorigenesis observed after a 20-fold higher γ-ray dose, i.e., 2 Gy, relative to the unirradiated group. Furthermore, previous studies have reported the role of GI epithelial cell senescence and the subsequent SASP signaling [17, 26], along with the upregulation of anti-apoptotic BCL-2 family proteins, including BCL-XL, during IR-induced cellular senescence and carcinogenesis [47]. IR-induced increase in adenoma and carcinoma frequency was accompanied by an increased number of BCL-XL-expressing senescent/SASP cells in the normal appearing intestinal mucosa, while similar BCL-XL protein expression patterns were observed in both spontaneous and 2 Gy-irradiated mouse tumor samples (Supplementary Figure 2). In concurrence, we observed that ABT-263 did not show a significant reduction in spontaneous adenoma and carcinoma frequency compared to the vehicle group, but it effectively decreased IR-induced adenoma and carcinoma frequency. This suggests that ABT-263’s mitigation of IR-induced GI tumorigenesis is linked to the elimination of BCL-XL-expressing senescent/SASP cells, as ABT-263 induces apoptosis in senescent cells by inhibiting BCL-XL [44].

Exposure to IR is known to increase the accumulation of senescent cells and the acquisition of a SASP in multiple tissues, including GI [17, 26]. Our finding of increased BCL-XL in a subset of IR-induced senescent intestinal cells is noteworthy because BCL-XL overexpression is known to promote the survival of senescent cells [48]. Additionally, ABT-263 shows the highest affinity for BCL-XL [44], which is the key target protein expressed in the mouse intestine (Supplementary Table 1). Therefore, its impact on non-senescent cells with higher BCL-XL expression is possible, but no apparent histological impact of ABT-263 was observed relative to vehicle only group. However, cells that do express high levels of BCL-XL proteins, such as senescent cells with anti-apoptotic phenotype (dual positive for p16/BCL-XL) and also tumor cells with higher BCL-XL, could potentially be eliminated by ABT-263. However, effective elimination of these cells requires further investigations as ABT-263 induced senolysis is likely to depend on several factors, including intrinsic resistance/sensitivity of a cell type, microenvironment, and drug bioavailability.

IR-induced increase in anti-apoptotic BCL-XL within the cells of the intestinal mucosa are likely to contribute to the persistence of cellular senescence in the GI tract. This could potentially lead to acquisition of SASP, which is associated with increased secretion of SASP factors, resulting in a pro-inflammatory microenvironment conducive to tumor development [42, 43]. ABT-263 is expected to reduce the senescent cell population with an anti-apoptotic phenotype (i.e., higher BCL-XL) and, therefore, could dampen SASP factors associated with a higher risk of tumor development. To support this, we identified six cytokines with established roles in GI cancer development (Supplementary Table 2) that were upregulated in serum after ^28^Si exposure and were effectively reversed by ABT-263. Moreover, some of these cytokines such as CXCL4, are also known to promote the production of IL6 and the acquisition of SASP [49, 50], potentially creating a feedback loop that drives chronic inflammation long-term after exposure to low- and high-LET radiation. While our results demonstrate a reduction in IR-induced serum SASP factors, the possibility that these factors are secreted by tumors still exists, as the serum analysis was conducted at only one time point, 5 months post-exposure. Since tumor burden is also reduced in the IR + ABT-263 group, and the SASP component measurements appear lower, further studies including a time-course and parallel assessment of tumor burden are needed to differentiate between the contributions from tumor-associated SASP and other sources of SASP after total body IR exposure.

IR-induced chronic inflammation is known to activate oncogenic β-catenin signaling, which is associated with a higher risk of GI carcinogenesis [12, 46, 51]. ABT-263 administration was found to significantly decrease the levels of active β-catenin and its downstream effector cyclin D1. Notably, a remarkable suppression of β-catenin and its downstream effector cyclin-D1 was noted in the irradiated mouse intestine. This suggests that ABT-263 has the potential to modulate this signaling pathway and mitigate GI carcinogenesis following both low- and high-LET IR exposure (Figure 6E). Our findings also corroborate earlier reports demonstrating ABT-263-mediated reduction in β-catenin and its downstream target cyclin D1 [52], highlighting the established role of this signaling pathway in IR-induced GI carcinogenesis in *Apc*-mutant mouse models [53].

In conclusion, this study serves as a proof of principle that targeting senescent cells using senotherapeutic agents, such as ABT-263, can effectively mitigate the risk of both low- and high-LET IR-induced GI tumor and carcinoma incidence. IR-triggered chronic inflammation, particularly in GI tissues, and ABT-263’s ability to modulate inflammatory cytokines and inhibitory effects on the β-catenin signaling pathway were noted. These findings suggest potential mechanisms by which ABT-263 mitigates the risk of IR-induced GI cancer development and highlights the importance of senolytic-based therapeutic interventions. While the findings highlight the potential of senolytic intervention in reducing cancer risk by eliminating senescent cells and associated pro-inflammatory signals, it’s important to note that ABT-263 may not be the ideal therapeutic agent due to its known side effects, particularly after long-term use, are associated with the risk of thrombocytopenia [54]. However, the strategy demonstrated here could be extended to other known senotherapeutic approaches, using novel BCL-2/BCL-XL degraders, D+Q (Dasatinib and Quercetin), Fisetin, and SASP-neutralizing monoclonal antibodies (SNmAbs) etc. [14, 16, 55]. Future research should focus on validating and optimizing these senotherapeutic strategies to achieve the desired safety without compromising their therapeutic index. This approach could be a viable option for preventing IR-induced cancer risk as well as the adverse health effects related to DEARE (delayed effects of acute radiation exposure).

## MATERIALS AND METHODS

### Animal maintenance and procedures

The *Apc*^1638N/+^ mouse (C57BL6 background) line was bred, genotyped, and maintained at the Georgetown University (GU) animal facility, as described earlier [56]. Throughout the study period, all animals were group housed (5 per cage) in ventilated cages with approved rodent diet and filtered water placed in a room with controlled humidity (50%), temperature (22° C), and ambient light (12-hour dark-light cycle). At eight to nine weeks of age, male *Apc*^1638N/+^ mice were randomly assigned to experimental groups and exposed to either sham or total-body low-LET γ-rays or high-LET ^28^Si-ion, as described previously [2, 12]. In brief, animals in the sham and irradiation groups were transported to the Brookhaven National Laboratory (BNL) in Upton, NY, and acclimatized for one week before irradiation. Animals were either irradiated with ^28^Si-ion (300 MeV/n, 69 keV/μm, mean dose rate ~0.1 Gy/min) at the National Aeronautics and Space Administration (NASA) Space Radiation Laboratory (NSRL) or with γ-rays (^137^Cs source, J.L. Shepherd and Associates Mark-I Model 68A Irradiator, dose rate ~0.97 Gy/min) at BNL. Sham animals were kept under similar conditions as the irradiation group animals. The day after irradiation, all mice were returned back to the GU animal facility via an approved same-day animal courier service and maintained there until the study endpoint.

Notably, the relative biological effectiveness (RBE) for tumorigenesis in *Apc*^1638N/+^ mice after a 0.1 Gy ^28^Si-ion was 18-fold higher (ranging from 12.9 to 26-fold) than that of low-LET IR [10]. Therefore, we selected a 2 Gy dose for γ-rays and a 0.1 Gy for ^28^Si-ion, as these doses are expected to result in similar tumor yield in *Apc*^1638N/+^ mice. Beginning eight weeks post-exposure, 50 mg/kg of ABT-263 (Cat # S1001, Selleck Chemicals LLC, Houston, TX, USA) dissolved in 30% polyethylene glycol, 60% phosal, and 10% ethanol, was administered through oral gavage (5 days/week) until euthanasia and tissue collection. The effect of oral ABT-263 on mouse body weight was also recorded and is presented in the Supplementary Figure 3. A schematic summary of irradiation, drug administration, and euthanasia is presented in Figures 2A, 4A. Animals were regularly monitored, and procedures, including irradiation, drug treatment, and euthanasia, were performed in accordance with the approved Institutional Animal Care and Use Committee (IACUC) protocol GU# 2016-1129, BNL# 345. All experimental animals were exposed to similar restraint and handling conditions until euthanasia.

### Euthanasia, sample collection, and histopathology

At five months post-exposure, all experimental mice were euthanized using a carbon dioxide (CO_2_) chamber with a controlled flow rate set at 30 to 60% of the cage volume per minute. Blood samples were collected via cardiac puncture, followed by the harvesting of GI tissues. Blood samples were directly collected in BD Microtainer serum separator tubes (product ref. no. 365967) and immediately processed for serum using the manufacturer’s recommended protocol. Aliquots of the serum samples were then flash-frozen and stored at -80° C until use. After appropriately cleaning the GI tissues with phosphate-buffered saline (PBS), the intestinal lumen was longitudinally opened, and GI tumors were scored using a dissecting microscope (Leica MZ6) by multiple observers who were blinded to the experimental groups. Finally, segments of normal and tumor-bearing GI tissue were fixed in buffered formalin and embedded in paraffin. Formalin-fixed paraffin-embedded (FFPE) GI tissues were cut using a microtome to obtain 4–6-micron thick sections, which were used for immunohistochemical staining. Hematoxylin and eosin (H&E)-stained tumor sections were evaluated by a board-certified pathologist to classify the tumors as either benign adenomas or invasive carcinomas. For each group, the percentages of adenomas and carcinomas relative to the total number of tumors assessed were calculated. The frequency of carcinomas was determined by dividing the number of carcinomas by the total number of tumors in each group, referred to as the total carcinoma frequency. The induced carcinoma frequency was calculated by subtracting the spontaneous carcinoma frequency (from the control or vehicle group) from the total carcinoma frequency in the irradiated groups.

### Immunofluorescence staining and quantification

FFPE tissue sections were deparaffinized and rehydrated, followed by antigen retrieval using citrate buffer (Electron Microscopy Sciences, Hatfield, PA, USA). After an appropriate blocking step, sections were incubated overnight with primary antibodies [anti-p16INK4a (1:1000 dilution, MAS-17142; Invitrogen, Carlsbad, CA, USA), anti-Bcl-XL (dilution 1:100; mAb #2764; Cell Signaling Technology, Danvers, MA, USA), IL6 (cat#ab7737; dilution-1:200; Abcam, Cambridge, MA, USA)] at 4-6° C. The next morning, sections were washed and incubated with Alexa Fluor 488 (green) or 546 (red) conjugated secondary antibodies for 2 h in the dark at room temperature. Finally, following multiple washing steps, sections were mounted using a 4′,6-diamidino-2-phenylindole (DAPI) containing mounting medium (Electron Microscopy Sciences, Hatfield, PA, USA) and immunofluorescence imaging was performed using an Olympus BX63 microscope equipped with a Hamamatsu digital camera (model# C11440-42U30). Immunofluorescence TIFF images were acquired at 400x magnification from the jejunal region (n=5 mice/group) at fixed microscopic acquisition settings for all the experimental groups using cellSens Entry v1.15 (Olympus Corp, Center Valley, PA, USA) software. The images were adjusted for the respective RGB color thresholds in FIJI (ImageJ) software and 8 to 12 regions of interest were identified. Cells exceeding the cutoff threshold for each marker (or a combination of markers) were counted as positive (or dual-positive) within each jejunal crypt [total 20 jejunal crypts (>1000 cells) from each experimental group] and were counted to evaluate the number of p16, BCL-XL, IL6, and dual marker positive (p16/BCL-XL or p16/IL6) cells using FIJI ImageJ2 software version 2.9.0/1.53t (fiji.sc). Additionally, the quantification of individual senescent (p16-positive) and SASP (IL6-positive) markers is presented in Supplementary Figure 4.

### Immunohistochemical staining and quantification

FFPE tissue sections were deparaffinized and rehydrated, followed by antigen retrieval using citrate buffer. After an appropriate blocking step, the tissue sections were incubated overnight with primary antibodies, namely anti-Active-β-Catenin [clone 8E7; Cat#05–665, dilution: 1:150; Millipore Sigma, Billerica, MA, USA] or anti-cyclin D1 [clone: EPR2241 (IHC)-32; Cat#04-1151; dilution: 1:150; Millipore]. The next morning, all sections were washed and protein expression was detected using 3,3’Diaminobenzidine (DAB)-based detection using the HRP/DAB IHC detection kit (Cat#ab236466, Abcam), following the manufacturer’s instruction. Finally, all sections were counterstained using Mayer’s hematoxylin (Electron Microscopy Sciences), mounted in Permount (Electron Microscopy Sciences), and subjected to bright field imaging using an Olympus BX63 microscope equipped with a DP28 camera and cellSens Entry v1.15 software (Olympus Corp, Center Valley, PA, USA). Eight to twelve randomly selected TIFF images were captured. In the case of active β-Catenin, quantification was performed based on DAB staining intensity. For cyclin D1, the number of positive nuclei in the jejunal crypt cells was quantified using the ITCN (Image-based Tool for Counting Nuclei) plugin in Fiji (ImageJ2) software. All data were presented as fold change relative to the control group.

### Mouse serum inflammatory cytokine analysis

Serum samples obtained at five months post-exposure were used to assess the relative levels inflammatory cytokines. The Mouse cytokine antibody array C3 (AAM-Cyt3, RayBiotech, Peachtree Corners, GA, USA) was used for the semi-quantitative assessment of cytokines in the mouse serum samples, following the manufacturer’s protocol. Details of all the analyzed cytokines are provided in Supplementary Figure 1, and mouse serum cytokines altered in response to ABT-263 are presented in Supplementary Table 3. In brief, the array membranes were first blocked with a blocking buffer and then incubated with two-fold diluted mouse serum samples for 1.5 hours. This was followed by washing and incubation with horseradish peroxidase (HRP)-labeled biotinylated secondary antibodies for another 1.5 hours at room temperature. Signals were detected using the electro-chemiluminescence (ECL) method and an ECL image scanner (GE, Amersham, UK). The scanned images were quantified using Fiji Image J2 software, and signal intensity was normalized with positive controls from the same array membrane for each group. Differential cytokines with a fold change >1.2 and p<0.05 were used to assess the effect of ABT-263. Finally, cytokine array data were used for principal component analysis and heatmap generation to illustrate groupwise differences using ClustVis 2.0 [57].

### BCL2 family gene transcript abundance analysis

For the basal expression analysis of BCL2 family mRNA in normal mouse and human small intestine, the RNA-seq databases [ENCODE transcriptome data (Bioproject PRJNA66167 for mouse) [58] and HPA RNA-seq normal tissue (Bioproject PRJEB4337 for human) [59] were accessed. RPKM values for BCL2, BCL-XL, and BCL-W were obtained, and BCL-XL was identified as the most abundant BCL2 family member in the small intestine (Supplementary Table 1).

### Statistical analysis

Non-parametric analysis was used to determine the equality of variance for the quantitative analysis of GI tumors. One-way ANOVA followed by Dunn’s multiple comparison test was employed to assess the statistical significance (*p*<0.05) among tumor data between the control and treatment groups. A two-tail paired Student t-test was used to evaluate statistical significance (*p*<0.05) for the IHC and IF staining. Each group’s values are displayed as a bar graph with the means, and SEM is presented as error bars. Statistically significant differences between groups were considered when the *p*-value was less than 0.05. GraphPad Prism software (La Jolla, CA, USA) was utilized for all statistical analyses.

### Data availability statement

All relevant data have been made available in this manuscript or as Supplementary Material.

## Supplementary Material

Supplementary Figures

Supplementary Tables
