# Supplementary material for: Senolytic agent ABT-263 mitigates low- and high-LET radiation-induced gastrointestinal cancer development in Apc1638N/+ mice
Source: Aging (Albany NY). 2025 Jan 8;17(1):97–115. doi: 10.18632/aging.206183 (PMC11810060; doi:10.18632/aging.206183)
Supplement: Supplementary Figures [file aging-17-206183-s001.pdf]

SUPPLEMENTARY FIGURES

A

	A	B	C	D	E	F	G	H	I	J	K	L	M	N
1 2	POS	POS	NEG	NEG	BLANK	Axl	CXCL13	TNFSF8	TNFRSF8	TNFRSF5	CRG2	CCL27	CXCL16	Eotaxin1
3 4	Eotaxin2	TNFSF6	CX2CL1	GCSF	GM-CSF	IFNg	IGFBP3	IGFBP5	IGFBP6	IL1a	IL1b	IL2	IL3	IL3Rb
5 6	IL4	IL5	IL6	IL9	IL10	IL12 P40/p70	IL12 p70	IL13	Il17A	CXCL1	LeptinR	Leptin	LIX	L-Selectin
7 8	XCL1	CCL2	MCP5	MCSF	CXCL9	CCL3	MIP1g	MIP2	CCL19	CCL20	CXCL4	P-Selectin	CCL5	SCF
9 10	SDF1a	CCL17	TCA3/CCL1	CCL25	TIPM1	TNFa	TNFRSF1A	TNFRSF1B	TPO	VCAM1	VEGFa	BLANK	BLANK	POS

B

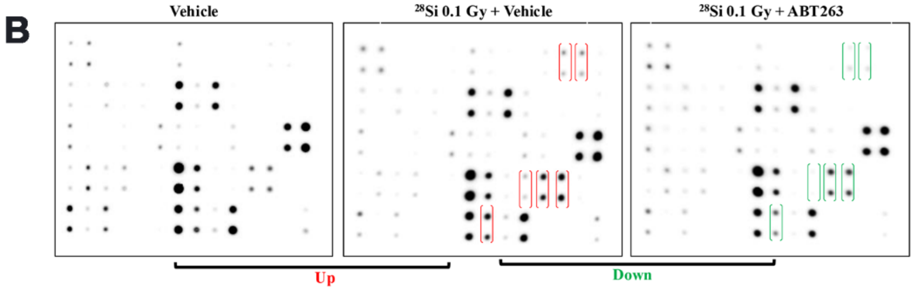

Supplementary Figure 1. Mouse cytokine array. (**A**) Spot map of mouse cytokine array C3 spot map (Cat#AAM-Cyt3; RayBiotech) in tabular form. Each antibody is spotted in duplicate as vertical dots. (**B**) Representative electrochemiluminescence (ECL) image depicting differential expression of serum cytokine levels in the respective groups. Highlighted proteins were upregulated after 28Si exposure and were mitigated using ABT-263.

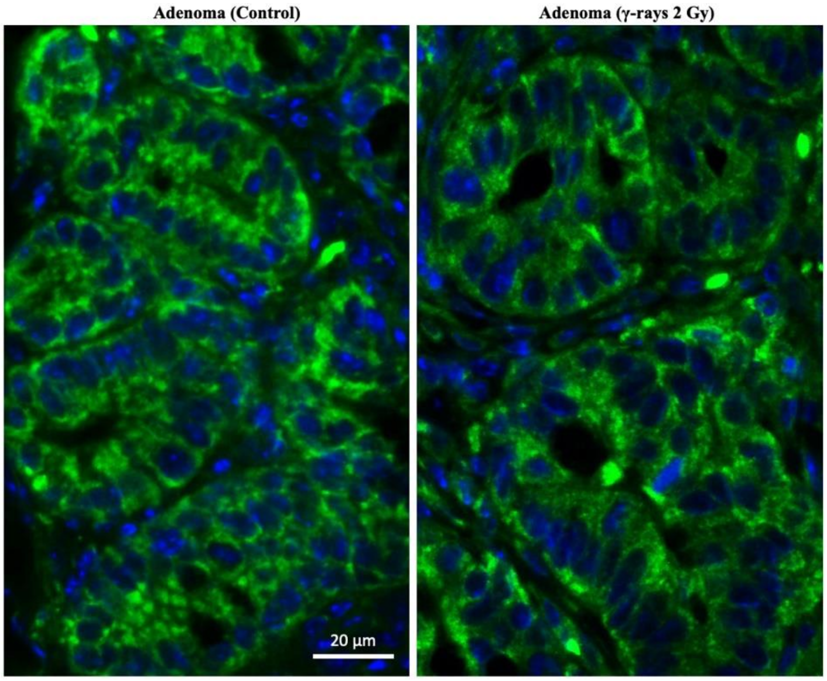

Supplementary Figure 2. Immunofluorescence-based assessment of BCL-XL (green color) protein expression in formalin-fixed paraffin embedded tumor tissue sections from control (spontaneous) and IR-exposed *Apc*1638N/+ mice.

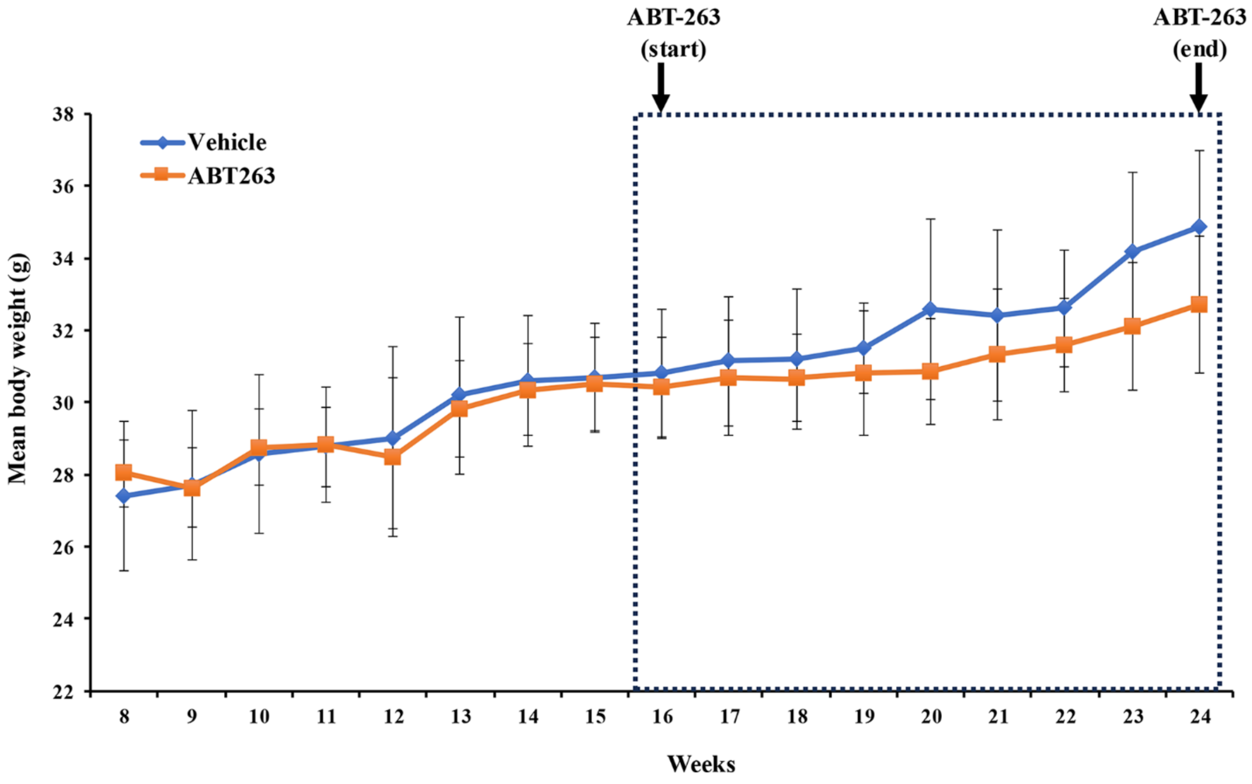

Supplementary Figure 3. Effect of ABT-263 on mouse body weight. Beginning sixteen weeks of age (marked by dotted box). ABT-263 was administered through oral gavage (5 days/week) until euthanasia and body weight was recorded weekly and presented as mean body weight in grams (g). Error bars are representing the standard deviation (SD) from the mean value.

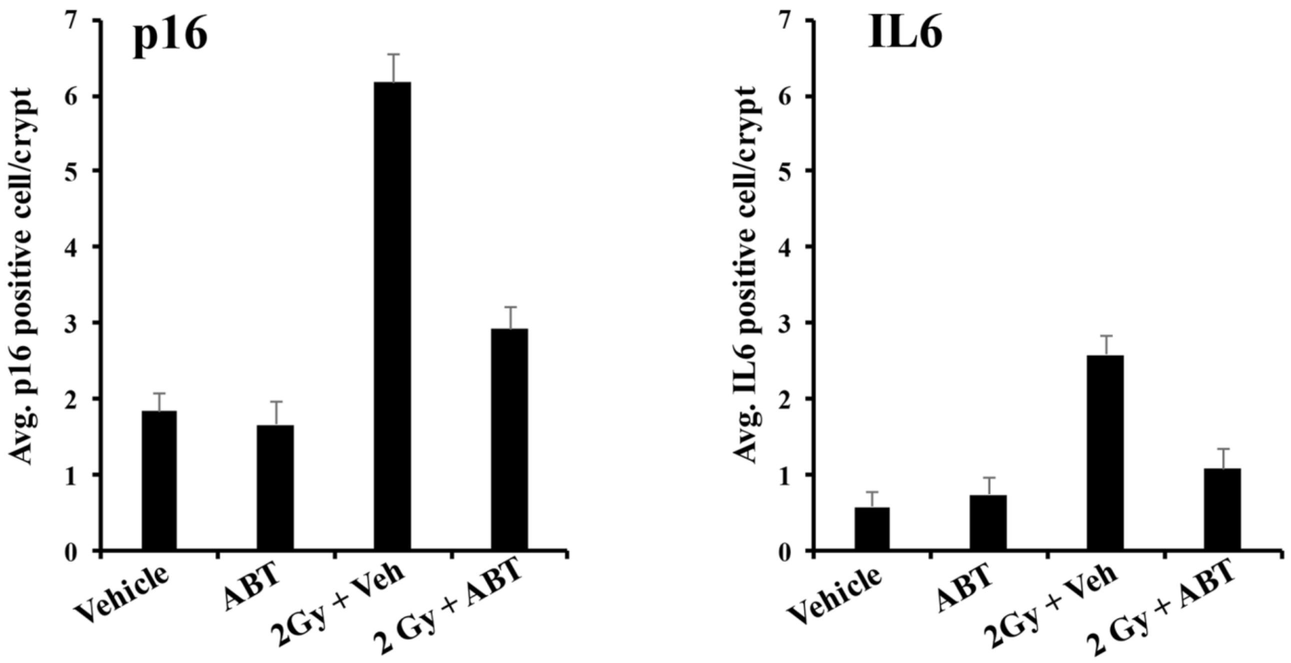

Supplementary Figure 4. Immunofluorescence-based quantification of senescent (p16 positive) and SASP (IL6 positive) cells in the *Apc*$^{1638N/+}$ mice jejunal crypts.
