# Supplementary material for: Senolytic agent ABT-263 mitigates low- and high-LET radiation-induced gastrointestinal cancer development in Apc1638N/+ mice
Source: Aging (Albany NY). 2025 Jan 8;17(1):97–115. doi: 10.18632/aging.206183 (PMC11810060; doi:10.18632/aging.206183)
Supplement: Supplementary Tables [file aging-17-206183-s002.pdf]

SUPPLEMENTARY TABLES

Supplementary Table 1. Basal mRNA expression [reads per kilobase per million mapped (RPKM) values] of ABT-263 target BCL2 family genes in mouse and human small intestine.

BCL family gene (symbol)	Mouse [1]	Human [2]
B-cell leukemia/lymphoma 2 *(Bcl2)*	0.381	2.13
Bcl2 like 1 *(Bcl2l1 or Bcl-xL)*	**4.549**	**16.92**
Bcl2 like 2 *(Bcl2l2 or Bcl-w)*	2.64	13.5

Supplementary Table 2. List of pro-inflammatory and GI cancer-associated serum factors upregulated after ^{28}Si exposure and mitigated using ABT-263.

Protein name (symbol)	Role in inflammation	Role in GI cancer development
Tumor Necrosis Factor Receptor Superfamily, Member 1B (TNFRSF1B)	Yes [3]	Yes [4]
Chemokine (C-C Motif) Ligand 20 (CCL20)	Yes [5]	Yes [5]
Chemokine (C-X-C Motif) ligand 4 (CXCL4)	Yes [6]	Yes [7, 8]
P-Selectin (SELP)	Yes [9]	Yes [10]
Chemokine (C-C Motif) Ligand 27 (CCL27)	Yes [11]	Yes [11]
Chemokine (C-X-C Motif) Ligand 16 (CXCL16)	Yes [12]	Yes [13, 14]

Supplementary Table 3. List of significantly altered serum cytokines in response to ABT-263.

Protein	Fold change (± SEM)	Regulation
AXL Receptor Tyrosine Kinase (Axl)	-1.27± 0.07*	Down
Tumor Necrosis Factor (ligand) Super Family Member 6 (TNFSF6)	-1.42±0.04*	Down
Fractalkine (CX2CL1)	-1.47±0.32*	Down
Granulocyte-Colony Stimulating Factor (G-CSF)	-1.40±0.04*	Down
Granulocyte-Macrophage Colony Stimulating Factor (GM-CSF)	-1.33 ±0.02*	Down
Interferon Gamma (IFN-gamma)	-1.21 ± 0.03*	Down
C-C Motif Chemokine Ligand 5 (CCL5)	1.24 ± 0.02*	Up
Tissue Inhibitor of Metalloproteinase 1 (TIMP1)	1.22 ± 0.02*	Up
Tumor Necrosis Factor Receptor Superfamily Member 1B (TNFRSF1B)	1.00 ± 0.008	Unchanged
C-C Motif Chemokine Ligand 20 (CCL20)	1.15 ± 0.03	Unchanged
Chemokine (C-X-C Motif) Ligand 4 (CXCL4)	1.09 ± 0.001	Unchanged
P-Selectin	-1.03 ± 0.07	Unchanged
C-C Motif Chemokine Ligand 27 (CCL27)	1.15 ± 0.11	Unchanged
C-X-C Motif Chemokine Ligand 16 (CXCL16)	1.20 ± 0.11*	Up

*Significantly altered in ABT-263 group relative to vehicle group [cut-off of 1.2-fold at *p*<0.05].

REFERENCES

1. Moore JE, Purcaro MJ, Pratt HE, Epstein CB, Shoresh N, Adrian J, Kawli T, Davis CA, Dobin A, Kaul R, Halow J, Van Nostrand EL, Freese P, et al, and ENCODE Project Consortium. Expanded encyclopaedias of DNA elements in the human and mouse genomes. Nature. 2020; 583:699–710. https://doi.org/10.1038/s41586-020-2493-4 PMID:32728249

2. Fagerberg L, Hallström BM, Oksvold P, Kampf C, Djureinovic D, Odeberg J, Habuka M, Tahmasebpoor S, Danielsson A, Edlund K, Asplund A, Sjöstedt E, Lundberg E, et al. Analysis of the human tissue-specific expression by genome-wide integration of transcriptomics and antibody-based proteomics. Mol Cell Proteomics. 2014; 13:397–406. https://doi.org/10.1074/mcp.M113.035600 PMID:24309898

3. Pan S, Liu R, Wu X, Ma K, Luo W, Nie K, Zhang C, Meng X, Tong T, Chen X, Wang X, Deng M. LncRNA NEAT1 mediates intestinal inflammation by regulating TNFRSF1B. Ann Transl Med. 2021; 9:773. https://doi.org/10.21037/atm-21-34 PMID:34268386

4. Zhao T, Li H, Liu Z. Tumor necrosis factor receptor 2 promotes growth of colorectal cancer via the PI3K/AKT signaling pathway. Oncol Lett. 2017; 13:342–6. https://doi.org/10.3892/ol.2016.5403 PMID:28123565

5. Olsen RS, Nijm J, Andersson RE, Dimberg J, Wågsäter D. Circulating inflammatory factors associated with worse long-term prognosis in colorectal cancer. World J Gastroenterol. 2017; 23:6212–9. https://doi.org/10.3748/wjg.v23.i34.6212 PMID:28974887

6. Silva-Cardoso SC, Tao W, Angiolilli C, Lopes AP, Bekker CP, Devaprasad A, Giovannone B, van Laar J, Cossu M, Marut W, Hack E, de Boer RJ, Boes M, et al. CXCL4 Links Inflammation and Fibrosis by Reprogramming Monocyte-Derived Dendritic Cells *in vitro*. Front Immunol. 2020; 11:2149. https://doi.org/10.3389/fimmu.2020.02149 PMID:33042127

7. Zhang Y, Gao J, Wang X, Deng S, Ye H, Guan W, Wu M, Zhu S, Yu Y, Han W. CXCL4 mediates tumor regrowth after chemotherapy by suppression of antitumor immunity. Cancer Biol Ther. 2015; 16:1775–83. https://doi.org/10.1080/15384047.2015.1095404 PMID:26479470

8. Ruytinx P, Proost P, Struyf S. CXCL4 and CXCL4L1 in cancer. Cytokine. 2018; 109:65–71. https://doi.org/10.1016/j.cyto.2018.02.022 PMID:29903575

9. Schürmann GM, Bishop AE, Facer P, Vecchio M, Lee JC, Rampton DS, Polak JM. Increased expression of cell adhesion molecule P-selectin in active inflammatory bowel disease. Gut. 1995; 36:411–8. https://doi.org/10.1136/gut.36.3.411 PMID:7535284

10. Qi C, Wei B, Zhou W, Yang Y, Li B, Guo S, Li J, Ye J, Li J, Zhang Q, Lan T, He X, Cao L, et al. P-selectin-mediated platelet adhesion promotes tumor growth. Oncotarget. 2015; 6:6584–96. https://doi.org/10.18632/oncotarget.3164 PMID:25762641

11. Song M, Sasazuki S, Camargo MC, Shimazu T, Charvat H, Yamaji T, Sawada N, Kemp TJ, Pfeiffer RM, Hildesheim A, Pinto LA, Rabkin CS, Tsugane S. Circulating inflammatory markers and colorectal cancer risk: A prospective case-cohort study in Japan. Int J Cancer. 2018; 143:2767–76. https://doi.org/10.1002/ijc.31821 PMID:30132835

12. Lehrke M, Konrad A, Schachinger V, Tillack C, Seibold F, Stark R, Parhofer IG, Broedl UC. CXCL16 is a surrogate marker of inflammatory bowel disease. Scand J Gastroenterol. 2008; 43:283–8. https://doi.org/10.1080/00365520701679249 PMID:18938659

13. AbdelMageed M, Ali H, Olsson L, Lindmark G, Hammarström ML, Hammarström S, Sitohy B. The Chemokine CXCL16 Is a New Biomarker for Lymph Node Analysis of Colon Cancer Outcome. Int J Mol Sci. 2019; 20:5793. https://doi.org/10.3390/ijms20225793 PMID:31752131

14. Korbecki J, Bajdak-Rusinek K, Kupnicka P, Kapczuk P, Simińska D, Chlubek D, Baranowska-Bosiacka I. The Role of CXCL16 in the Pathogenesis of Cancer and Other Diseases. Int J Mol Sci. 2021; 22:3490. https://doi.org/10.3390/ijms22073490 PMID:33800554
